# The role of vitamin K2 in cognitive impairment: linking vascular health to brain health

**DOI:** 10.3389/fnagi.2024.1527535

**Published:** 2025-01-15

**Authors:** Stefanos Roumeliotis, Ioannis Kontogiorgos, Femke de Vries, Katarzyna Maresz, Jean-François Jeanne, Konstantinos Leivaditis, Leon J. Schurgers

**Affiliations:** ^1^Second Department of Nephrology, School of Medicine, AHEPA Hospital, Aristotle University of Thessaloniki, Thessaloniki, Greece; ^2^Department of Biochemistry, Cardiovascular Research Institute Maastricht, Maastricht, Netherlands; ^3^International Science & Health Foundation, Krakow, Poland; ^4^Gnosis by Lesaffre, Lesaffre International, R&D Department, Marcq-En-Baroeul, France

**Keywords:** vitamin K2, menaquinone-7, arterial calcification, arterial stiffness, dementia, cognitive function

## Abstract

Cognitive impairment, marked by a decline in essential mental aspects such as attention, memory, and problem-solving, is significantly correlated with advancing age. This condition presents a major challenge for the elderly, adversely affecting quality of life, diminishing independence, and imposing substantial burdens on healthcare systems. Recent research indicates that vitamin K2 may be vital for preserving brain health and cognitive function. Traditionally recognized primarily for its role in blood coagulation, vitamin K has emerged in recent years as a nutrient with diverse biological effects essential for healthy aging. A growing body of evidence from both observational and interventional studies underscores the pivotal role of vitamin K2 in mitigating arterial calcification. This mechanism may link vascular health to cognitive function, suggesting that vitamin K2 could play a critical role in the prevention of cognitive impairment in aging populations.

## Introduction

By 2050, the World Health Organization (WHO) projects that the global population of individuals aged 80 and older will triple, leading to a significantly aged population worldwide (Ageing and Health, 2024). Since aging is usually accompanied by heavy mortality and comorbidity which burdens the economic healthcare systems, recently, healthy aging has been in the center of scientific research. Cognitive impairment, termed as decline in mental status and abilities including attention, memory, and problem-solving, is a condition tightly and bidirectionally associated with advanced age. Decline in cognitive function is a major concern for the elderly, because it might affect quality of life and lead to loss of independence and burden health care systems. Therefore, it soon became evident that early detection of modifiable risk factors for cognitive decline is of utmost importance. Among these factors, during recent years, accumulating evidence suggest that dietary components, like nutritional vitamin K might play pivotal role in brain function and cognitive function.

Cardiovascular disease (CVD) is the leading cause of morbidity and mortality worldwide (Mensah et al., 2019). Previous research has established a link between vascular remodeling, atherosclerosis and brain function, and it is now widely recognized that CVD is negatively correlated with cognitive health (Alosco and Hayes, 2015; Dolan et al., 2010; Rusanen et al., 2014). Arterial stiffness, in particular, appears to be a critical factor in the functional and structural brain changes associated with aging (Vlachopoulos et al., 2010). Previous studies have demonstrated that arterial stiffness contribute to cognitive decline through impaired cerebral perfusion. Given these, arterial stiffness has emerged as a potential therapeutic target to mitigate cognitive decline, highlighting the importance of early vascular interventions in preserving cognitive health.

In this article, we review the association between vitamin K and cerebral function, discussing novel developments regarding its therapeutic role in arterial stiffness and cognitive health.

## The link between arterial stiffness and cognitive status

As arterial stiffness increases with age, the pulsatile load transmitted to peripheral arteries becomes abnormal, resulting in end-organ damage. The brain, with its high-flow, low-resistance vascular system, is particularly vulnerable to this damage (Thorin-Trescases and Thorin, 2016). Arterial stiffness has been associated with age-related cerebral changes, such as atrophy and small vessel disease (Townsend et al., 2015; Laurent and Boutouyrie, 2020). Among the non-invasive measures of vascular stiffness, pulse wave velocity (PWV) is considered the gold standard. PWV measures arterial stiffness along the entire aortic pathway, providing a reliable, feasible, and accurate assessment of vascular health (Laurent et al., 2006; Boutouyrie et al., 2014). Increased arterial stiffness leads to impaired cerebral blood flow and white matter hypoperfusion, contributing to white matter (WM) structural abnormalities and white matter hyperintensities (WMH) (Jefferson et al., 2018; Promjunyakul et al., 2016). Moreover, recent studies have demonstrated that arterial stiffness, as measured by PWV, is negatively associated with total brain volume, brain atrophy, and cognitive function (Palta et al., 2019; Haidegger et al., 2023; Tomoto et al., 2023; Coffin et al., 2022).

In the Atherosclerosis Risk in Communities–Neurocognitive Study, the authors measured cognition status and performed brain magnetic resonance imaging to identify cerebral microbleeding, lacunar infarcts, white matter hyperintensities' volumes, and the signature pathognomonic region for Alzheimer's disease in a big cohort if older adults, aged 67–90 years (Palta et al., 2019). When patients were stratified according to their cfPWV and central pulse pressure values, the authors found that those with impaired central arterial hemodynamics presented with increased white matter hyperintensities, reduced total brain volumes and significantly poorer scores for general cognition, processing speed and executive function (Palta et al., 2019). This tight association between central arterial stiffness and brain structure impairment (assessed by reduced total brain volume, increased brain white matter hyperintensity volume and brain atrophy), is independent of gender and age (Tomoto et al., 2023) and of BP values and variability (Haidegger et al., 2023). Even in a diverse population of 460 people with a mean age of 70 ± 8 years with normal cognitive ability, mild impairment or dementia, surrogate markers of arterial stiffness were strongly associated with brain macro- and micro-structure impairment and both executive and global cognitive function decline. Specifically, cfPWV was an independent predictor of both high brain white matter volumes and diffusion based free water volumes (Coffin et al., 2022).

Several studies have proposed a causal relationship between arterial stiffness and cognitive impairment (Beulens et al., 2010; Rahimi Sakak et al., 2021; Tarkesh et al., 2020), with the most robust evidence emerging from meta-analyses. Pase et al. conducted a meta-analysis of six longitudinal studies involving 3,947 participants, finding that PWV is predictive of cognitive decline, as measured by the Mini-Mental State Examination (MMSE; β= −0.03, 95% CI: −0.06 to 0.01) (Pase et al., 2012). Van Stolen et al. performed a systematic review and meta-analysis to explore the association between arterial stiffness, microvascular cerebral disease (assessed by brain MRI), and cognitive function (van Sloten et al., 2015). While this study demonstrated a positive association between arterial stiffness and microvascular cerebral disease (OR: 1.39, 95% CI: 1.21–1.60), it could not definitively establish a correlation between PWV and cognitive impairment due to significant heterogeneity among the included studies (van Sloten et al., 2015). The primary limitations of these meta-analyses are the variability and quality of the studies involved.

To address these limitations, more recent meta-analyses have provided a comprehensive examination of the association between arterial stiffness and cognitive function over a long period. Alvarez-Bueno et al. conducted a meta-analysis of 38 studies (29 cross-sectional and nine longitudinal), including 43,115 participants, and assessed the relationship between arterial stiffness and specific cognitive indices, such as global cognition, executive function, and memory (Alvarez-Bueno et al., 2020). The results indicated that PWV was inversely correlated with global cognition (adjusted pooled ES = −0.21, 95% CI: −0.30 to −0.11), executive function (adjusted pooled ES = −0.08, 95% CI: −0.14 to −0.03), and memory (adjusted pooled ES = −0.13, 95% CI: −0.20 to −0.05) (Alvarez-Bueno et al., 2020). For the first time, this meta-analysis provided pooled estimates of the tight relationship between arterial stiffness and various cognitive functions. Another meta-analysis, spanning studies from January 1986 to March 2020, conducted separate qualitative and quantitative assessments of both cross-sectional and longitudinal studies (Liu et al., 2021). The cross-sectional analysis revealed a strong, negative association between memory, processing speed, and aortic PWV, while the longitudinal analysis found that participants in the high PWV category had a 44% higher risk of cognitive decline compared to those in the low category (OR: 1.44; 95% CI: 1.24–1.85) (Liu et al., 2021). Moreover, for every 1 m/s increase in aortic PWV, there was a 3.9% increase in the risk of cognitive impairment (OR: 1.039; 95% CI: 1.005–1.073) (Liu et al., 2021). The meta-regression analysis further indicated that the association between arterial stiffness and cognitive impairment intensified with age (Liu et al., 2021).

Traditionally, it has been proposed that intimal arterial calcification leads to obstruction of the artery and plaque rupture, whereas, calcification of the media might lead to arterial stifness, systolic hypertension and high PWV, which in turn trigger diastolic dysfunction and eventually heart failure. Arterial calcification is a major determinant of stiffening the arteries, which is considered the hallmark of vascular aging and an independent predictor if cardiovascular diseases. The pathogenetic mechanisms responsible for vascular stifness recently shifted from collagen and elastin to the differentiation of vascular smooth muscle cells to osteoblastic phenotype, which is triggered by oxidative stress and inflammation, membrane mechanotransduction, lipid metabolism, genetic factors and epigenetics (Lacolley et al., 2020).

Cognitive dysfunction, which is prevalent in aging populations, may be partially explained by increased arterial stiffness and calcification.

## Vitamin k

Vitamin K is a family of fat-soluble compounds, including vitamin K1 (phylloquinone) and vitamin K2 (menaquinones), which are structurally similar. While traditionally recognized for its role in blood clotting, recent studies have expanded the clinical relevance and understanding of vitamin K and now include bone and vascular health. Vitamin K-dependent proteins (VKDPs) rely on vitamin K to undergo γ-glutamylcarboxylation, a modification essential for their biological activity. This family of proteins includes hepatic VKDPs such as prothrombin, FVII, FIX, and FX, protein S and protein C as well as extrahepatic VKDPs such as matrix Gla-protein (MGP), which is involved in inhibiting vascular calcification, and osteocalcin, which plays a role in bone mineralization.

Vitamin K1, predominantly found in plant-based foods like leafy green vegetables, is the primary dietary form of vitamin K. Vitamin K2, however, is mainly produced by bacteria and is present in animal-derived foods and fermented products such as natto (fermented soy) and cheese. The structural differences between K1 and K2 influence their bioavailability, absorption, bioactivity, and distribution within tissues (Halder et al., 2019). Compared to vitamin K1, the K2 subtype menaquinone-7 (MK-7) has a significantly longer half-life, accumulates more effectively in blood, and exhibits greater biological activity, particularly in facilitating the carboxylation of extrahepatic VKDPs (Schurgers et al., 2007b). Currently, the daily recommended intake (DRI) for vitamin K is based solely on its role in blood coagulation and focuses primarily on preventing hemorrhaging. However, there is no established consensus on the optimal intake of vitamin K2, particularly with respect to its broader roles in bone, vascular, and cognitive health (Akbulut et al., 2020; Neofytou et al., 2024).

Recent research suggests that adequate vitamin K intake is necessary for activating extrahepatic VKDPs, which are critical for long-term health and cognitive function (Popescu and German, 2021). Circulating dephosphorylated, uncarboxylated Matrix Gla protein (dp-ucMGP), a marker of extrahepatic vitamin K deficiency, could represent a novel therapeutic target for mitigating both arterial stiffness and cognitive decline. MK-7 supplementation has been shown to be safe and well-tolerated, with no serious adverse events, and has demonstrated efficacy in improving vitamin K status in the elderly. Furthermore, evidence from clinical trials suggests that MK-7 may delay or even reverse vascular calcification (Knapen et al., 2015; Mansour et al., 2017; Eelderink et al., 2023; Naiyarakseree et al., 2023; Kurnatowska et al., 2015; Lees et al., 2019; Li et al., 2023).

## Evaluation of vitamin K

Assessing vitamin K intake and status in both population and clinical studies presents several challenges, as a range of methods are employed, each with limitations (Akbulut et al., 2020). The food frequency questionnaire (FFQ) is commonly used but is often inadequate for evaluating vitamin K2 intake due to incomplete food composition databases. Biomarkers that reflect vitamin K intake, absorption, and metabolism provide a more comprehensive assessment than dietary questionnaires. However, there is no single gold-standard test for vitamin K status. Various VKDPs, such as uncarboxylated prothrombin (PIVKA-II), the ratio of uncarboxylated to carboxylated osteocalcin (ucOC/cOC), and desphosphorylated-uncarboxylated MGP (dp-ucMGP), serve as markers of vitamin K status and are linked to diverse health outcomes. Recent studies have also linked these biomarkers to cognitive decline. Given the distinct functions of these biomarkers, employing multiple markers or combining them with dietary intake data could enhance the precision of vitamin K status evaluation (Shea and Booth, 2016).

## The role of vitamin K in brain function

Beyond its well-established role in haemostasis, emerging evidence suggests that vitamin K plays a critical role in brain function. Vitamin K affects cognitive and neural processes through various mechanisms, including the activity of several VKDPs (Table 1). Notably, growth arrest-specific 6 (Gas6) regulates cell survival and myelination (Binder et al., 2011; Varnum et al., 1995; Gilchrist et al., 2020), protein S mediates neuroprotective and antithrombotic effects by modulating the blood-brain barrier (Zhu et al., 2010) and MGP, a potent inhibitor of vascular calcification, has been linked to cognitive performance (Shea et al., 2022).

**Table 1:** VKDPs connected with the brain function.

**VKD**	**Involved in**	**References**
Gas6	Cell survival and growth, chemotaxis, mitogenesis, and myelination	Binder et al., 2011; Varnum et al., 1995; Gilchrist et al., 2020
Protein S	Antithrombotic and signaling-mediated neuroprotective actions	Zhu et al., 2010
	Modulation of the blood–brain barrier	
Protein C	Antithrombotic, anti-inflammatory, anti-apoptotic, and cell-signaling activities	Griffin et al., 2016
Osteocalcin	Brain development	Oury et al., 2013
	Production of several neurotransmitters that favor learning and memory formation	Obri et al., 2018
MGP	Inhibition of vascular calcification	Schurgers et al., 2008; Santa-Maria et al., 2010

In addition to influencing VKDPs, vitamin K exerts antioxidant effects by reducing levels of reactive oxygen species (ROS), with MK-7 being particularly effective in this regard (Mukai et al., 1993; Muszyńska et al., 2020; Shandilya et al., 2021). Given the link between oxidative stress, inflammation, and cognitive decline in the elderly (Baierle et al., 2015), the anti-inflammatory and antioxidant properties of vitamin K offer a promising prevention strategy (Huang et al., 2021; Yu et al., 2016). It is well established that vitamin K exerts beneficial effects on glucose homeostasis. Although the majority of studies focused on phylloquinone, MK-7 has also shown promising results in experimental studies and clinical trials. The large community-dwelling, prospective Dutch study in 38,094 participants showed that only dietary menaquinones were strongly and inversely associated with the risk for developing incident type 2 diabetes, during a long follow-up of 10 years; the association with dietary vitamin K1 was marginally lost (Beulens et al., 2010). Moreover, an RCT showed that daily 360 μg of MK-7 for 12 weeks in insulin-dependent patients with diabetes improved significantly fasting glucose levels, glycated hemoglobin, insulin concentrations and homeostatic model assessment for insulin resistance (HOMA-IR). Moreover, compared to the placebo, the MK-7 group had significantly higher number of patients that achieved optimum glycemic control (assessed by HbA1c and plasma glucose levels within target values) (Rahimi Sakak et al., 2021). Similar results were reported by another RCT in polycystic ovary syndrome patients; even low dose of MK-7 (90 μg daily for 8 weeks) significantly reduced serum fasting insulin, homeostatic model assessment for insulin resistance and β-cell function index, increased insulin sensitivity check index, improved lipid profile parameters and decreased waist circumference and body fat mass (Tarkesh et al., 2020). Taken together, these data highlight the beneficial effects of vitamin K (especially MK-7) on glycemic homeostasis, lipid metabolism and fat mass distribution. Maintaining optimum glucose metabolism is of utmost importance of brain cell function and viability and on the other hand, impaired glucose homeostasis occurs even at early stages of degenerative neurological conditions, including Parkinson's and Alzheimer's disease and contributes to the progression of these diseases (Camandola and Mattson, 2017; Putzu et al., 2018). This might be explained by the fact that glucose metabolism affects various factors that trigger brain aging, including glucose transport, nucleic acid repair, mitochondrial function and gut microbiome, which is the backbone of the gut-brain bidirectional axis (Wachsmuth et al., 2022). Therefore, MK-7, by regulating glucose metabolism might affect indirectly, brain structure and function.

Vitamin K also influences sphingolipid metabolism, which plays a critical role in neuronal function by regulating enzymes involved in sphingolipid biosynthesis (Alessenko and Albi, 2020; Denisova and Booth, 2005). Notably, certain functions of vitamin K2, such as its role as a mitochondrial electron carrier and transcriptional regulator via the steroid and xenobiotic receptor (SXR), are unique compared to vitamin K1 (Horie-Inoue and Inoue, 2008; Lin et al., 2021; Tang et al., 2022). While mitochondrial dysfunction is strongly associated with cognitive decline (Apaijai et al., 2020), further research is needed to establish whether improved mitochondrial function correlates with cognitive enhancement in humans.

## Experimental studies

**Figure 1:**
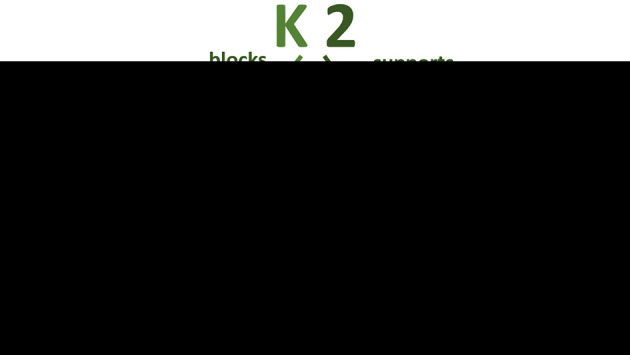
Multiple beneficial for brain actions of vitamin K2.

Animal studies have demonstrated that vitamin K deficiency leads to decreased levels of menaquinone-4 and sphingolipids in the brain, which may impair cognition and behavior (Tamadon-Nejad et al., 2018). Given the detrimental impact of suboptimal vitamin K status on the nervous system, it has been hypothesized that vitamin K2 supplementation could benefit brain function and cognition. In aged rats, MK-7 treatment reversed age-related cognitive deficits, improved inflammatory markers and redox balance, inhibited cerebrovascular calcification, and increased Gas6 protein expression, which was accompanied by cognitive improvements (Elkattawy et al., 2022; Lee et al., 2022). Another potential mechanism linking vitamin K deficiency to cognitive decline involves gut microbiome alterations. Inadequate vitamin K intake has been associated with significant changes in gut microbial composition (Tooley, 2020; Ellis et al., 2021) and animal models have demonstrated that vitamin K2 supplementation can improve cognitive outcomes by modulating the gut-brain axis (Chatterjee et al., 2023). These findings suggest that vitamin K2 has a multifaceted potential to enhance cognitive function, as illustrated in Figure 1.

## Clinical studies

The hypothesis that vitamin K depletion may adversely affect brain function is supported by clinical evidence, primarily derived from observational studies. A meta-analysis of eight studies involving 97,595 patients revealed that, compared to traditional long-term antithrombotic therapy with vitamin K antagonists (VKAs), which significantly reduce overall vitamin K status, the use of novel oral anticoagulants (NOACs) may reduce the risk of cognitive impairment (Zhang et al., 2018). Moreover, community-based studies have demonstrated that higher vitamin K intake is associated with reduced cognitive decline in the elderly (Chouet et al., 2015; Presse et al., 2008), and increased circulating vitamin K levels are linked to improved cognitive function (Presse et al., 2013), suggesting a potential preventive role against cognitive deterioration. Although multiple observational studies have examined the relationship between cognitive status, vitamin K-dependent biomarkers, and vitamin K2 concentrations in the brain, the results have been inconsistent, highlighting inherent challenges in this research area (Shea et al., 2022; Kiely et al., 2020; Ross et al., 2022; van den Heuvel et al., 2015).

There is accumulating data suggesting that increased dietary vitamin K intake abrogates the progression of cognitive decline in older adults and increased plasma/serum markers of vitamin K status are correlated with improved cognitive function. Since vitamin K is mainly found in leafy vegetables, this association could be an epiphenomenon of healthy diet and lifestyle. Moreover, the data regarding the types and quantity of vitamin K in the brain and their clinical significance are scarce. To test the research hypothesis that brain vitamin K levels might be associated with cognitive impairment and dementia, Booth et al., quantified vitamin K levels in regions of the human brain and examined their possible association with ante-mortem tests of cognitive ability and post-mortem neuropathologic findings in 325 decedents that participated in the Rush Memory and Aging Project (MAP). Apolipoprotein E genotypes were determined and K1 and MK-4 levels were evaluated in 4 different brain regions. Compared to phylloquinone, vitamin K2 (specifically MK-4) was the predominant form of vitamin K in all brain regions. Increased brain MK-4 levels were linked to decreased odds of mild cognitive decline, dementia, Braak stage, Alzheimer's disease scores and less neurofibrillary tangle density (Booth et al., 2022). Moreover, higher pro-mortem vitamin K1 was related with better cognitive function test scores and delayed progression of cognitive decline (Booth et al., 2022). These findings support the hypothesis that vitamin K2 plays a significant role in brain health. Although preliminary, these insights into the connection between vitamin K2 and cognitive performance in human studies offer intriguing avenues for further investigation (Booth et al., 2022).

## Vitamin k2 and cardiovascular health—evidence from interventional studies

**Figure 2:**
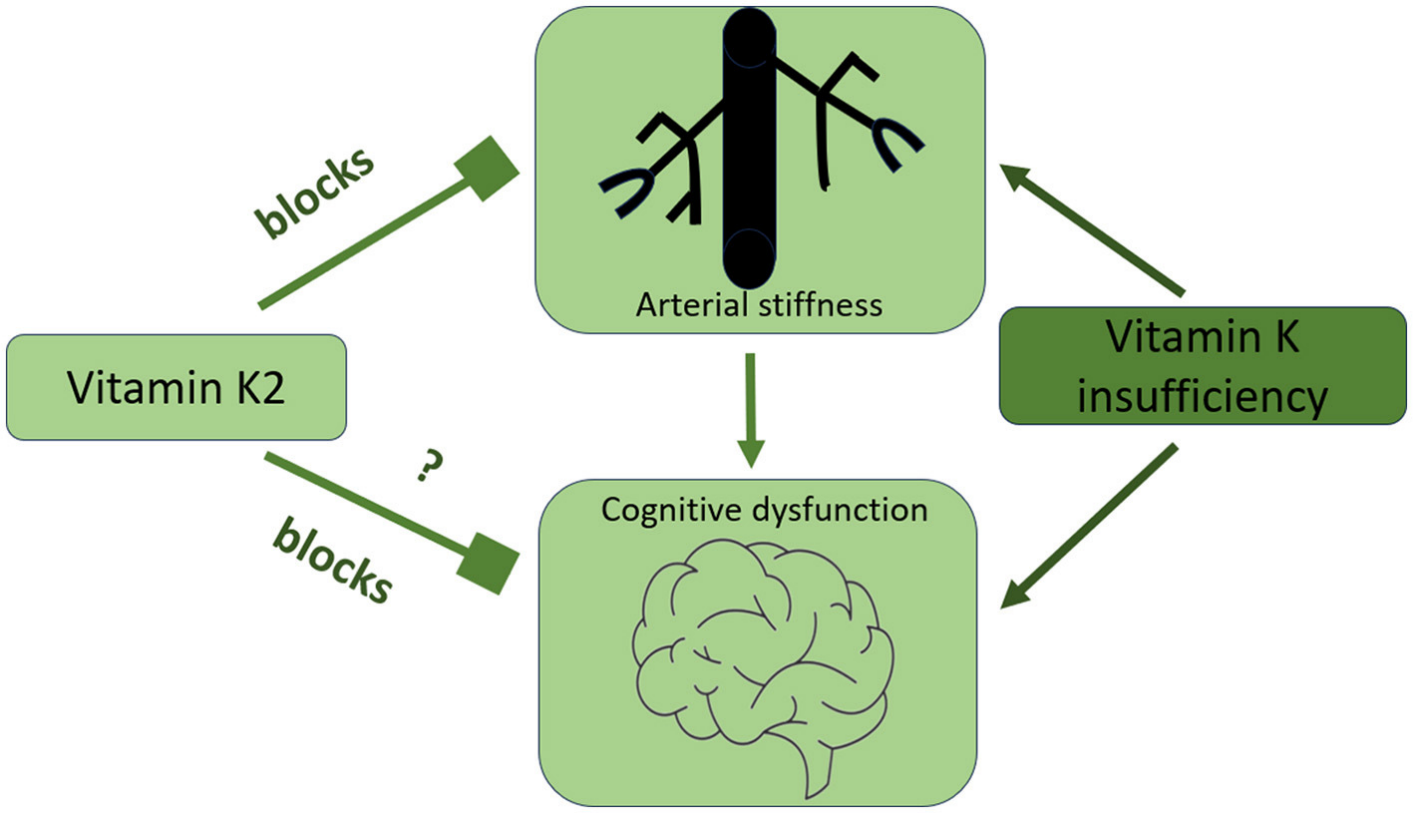
The connection between arterial stiffness, cognitive function, and optimal vitamin K intake.

Arterial stiffness is a dynamic process regulated by various proteins and molecules. Among these, matrix Gla protein (MGP) is the most potent tissue inhibitor of arterial stiffness and calcification. Experimental data suggest that vitamin K2, by activating MGP, may prevent vascular calcification and arterial stiffness, and support cardiovascular health (Spronk et al., 2003; Schurgers et al., 2007a; Scheiber et al., 2015). Based on these findings, a growing number of clinical studies have been conducted to investigate the effects of vitamin K2 in high-risk populations, such as patients with chronic kidney disease (CKD), end-stage kidney disease (ESKD), and diabetes. However, clinical research specifically evaluating the effects of vitamin K2 on arterial stiffness and cognitive performance remains limited (Figure 2). These emerging findings warrant further exploration, particularly through well-designed interventional studies, to fully elucidate the role of vitamin K2 in both cardiovascular and cognitive health.

Accumulating evidence suggests that dp-ucMGP is associated with several surrogate markers of arterial calcification (Roumeliotis et al., 2019; Hariri et al., 2021). Additionally, clinical data from studies in patients with impaired kidney function suggest that elevated plasma dp-ucMGP levels serve as independent predictors of adverse clinical outcomes, including all-cause and cardiovascular mortality (Kaesler et al., 2021; Keyzer et al., 2015; Roumeliotis et al., 2020, 2022a). The Rotterdam study was among the first to investigate the association between dietary vitamin K intake and cardiovascular events and all-cause mortality in a large cohort of 4,807 subjects without pre-existing cardiovascular disease (CVD) at baseline (Geleijnse et al., 2004). This landmark study demonstrated that only vitamin K2 (menaquinones) was inversely associated with coronary heart disease incidence, aortic calcification and mortality whereas vitamin K1 failed to show association with any of the outcomes. Moreover, vitamin K2 intake was negatively correlated with all-cause mortality and severe aortic calcification, underscoring the pivotal role of vitamin K consumption in cardiovascular disease prevention (Geleijnse et al., 2004).

Following the recognition of dp-ucMGP as an independent predictor of vascular calcification (VC), several studies explored its association with cardiovascular morbidity and mortality. Findings indicate that elevated dp-ucMGP levels are linked to adverse cardiovascular outcomes and mortality in both the general population (van den Heuvel et al., 2014; Liu et al., 2015) and in specific patient groups, including individuals with diabetes (Dalmeijer et al., 2013; Liabeuf et al., 2014), chronic kidney disease (CKD) (Schurgers et al., 2010; Roumeliotis et al., 2017) and those with pre-existing cardiovascular conditions (Ueland et al., 2010; Capoulade et al., 2014; Mayer et al., 2016).

Given this scientific background, numerous randomized controlled trials (RCTs) have been conducted to investigate the potential effects of vitamin K2, specifically menaquinone-7 (MK-7), on the progression of vascular calcification and cardiovascular disease prevention. One of the earliest double-blind RCTs investigating MK-7 supplementation in 244 postmenopausal women found that long-term (3-year) MK-7 administration of 180 μg/day improved arterial stiffness, as measured by the stiffness index (SI β; MK-7 group: −0.67 ± 2.78 vs. placebo group: +0.15 ± 2.51, *p* = 0.018) (Knapen et al., 2015). The beneficial effects were particularly pronounced in participants with increased baseline vascular stiffness (Knapen et al., 2015).

The KING trial (Vitamin K2 in Renal Graft), a single-center study involving 60 stable kidney transplant recipients, demonstrated that daily supplementation with MK-7 (360 μg/day) for 8 weeks resulted in a 14.2% reduction in carotid-femoral pulse wave velocity (cfPWV), a measure of arterial stiffness (pre-VK: 9.8 ± 2.2 m/s vs. post-VK: 8.4 ± 1.5 m/s, *p* < 0.001), reflecting a 40% improvement in subclinical vitamin K deficiency (Mansour et al., 2017). Similarly, another RCT in 40 kidney transplant recipients showed that 12 weeks of MK-7 supplementation (360 μg/day) led to a significant reduction in dp-ucMGP levels and arterial stiffness [MK-7 group: Δdp-ucMGP −385 (−631 to −269) pmol/L vs. placebo group: +39 (−188 to +183) pmol/L, *p* < 0.001] (Eelderink et al., 2023).

A notable study conducted by Naiyarakseree et al. evaluated the effects of oral MK-7 (375 μg once daily) for 24 weeks in 96 hemodialysis patients with pre-existing arterial stiffness, demonstrating that MK-7 reduced cfPWV in diabetic patients [−10.0% (95% CI: −15.9 to −0.8) vs. placebo: +3.8% (95% CI: −5.8 to +11.6), *p* = 0.008] (Naiyarakseree et al., 2023). A meta-analysis of 14 longitudinal studies, encompassing 10,726 patients and 13 RCTs involving 2,162 participants, found that vitamin K supplementation decreased arterial calcification by 9.1% (95% CI: −17.7 to −0.5, *p* = 0.04) through a 44.7% improvement in vitamin K status (95% CI: −65.1 to −24.3, *p* < 0.0001). Moreover, vitamin K-dependent proteins were strongly associated with reductions in cardiovascular disease and mortality (HR: 0.45, 95% CI: 0.07 to 0.83, *p* = 0.02) (Lees et al., 2019).

While MK-7 has proven to be the most bioactive and clinically relevant form of vitamin K, some studies have investigated the effects of vitamin K1 supplementation on vascular calcification and cardiovascular health. These studies suggest that vitamin K1 reduces vascular calcification (Brandenburg et al., 2017; Saritas et al., 2022). However, it is important to note that once vitamin K1 enters the body, it is partially converted to MK-4 in the intestines and other tissues, implying that the clinical effects of phylloquinone may be mediated via its transformation into MK-4.

However, when K1 enters the human body, it is converted to K2 in the intestine and other tissues and thus, the clinical effects of phylloquinone are exerted through transformation to K2.

Despite promising results, some RCTs, particularly in CKD patients, have yielded negative outcomes regarding the effects of vitamin K supplementation on arterial stiffness and cardiovascular risk. Trials such as the Trevasc-HDK study (Haroon et al., 2023), K4Kidneys study (Witham et al., 2020), ViKTORIES study (Lees et al., 2021), Valkyrie study (De Vriese et al., 2020), RenakVit trial (Levy-Schousboe et al., 2021) and the study by Oikonomaki et al. (2019). reported no significant improvement in cardiovascular outcomes. However, these studies suffered from various limitations, including small sample sizes, high dropout rates, and suboptimal vitamin K dosing (360 μg MK-7, 3 times/wk in the Trevasc-HDK, 400 μg/day m-7 in the K4Kidneys, menadiol diphosphate 5 mg, thrice weekly in the ViKTORIES, vitamin K2 2000 μg thrice weekly in the Valkyrie, 360 μg MK-7 daily in the RenakVit, 200 μg MK-7 daily in the Greek study). For example, in the RenakVit trial, only 21 patients completed the study (Levy-Schousboe et al., 2021). The ViKTORIES study raised concerns about the methods used to assess vitamin K deficiency, specifically regarding the accuracy of dp-ucMGP assays (Te Velde-Keyzer and de Borst, 2022) and the Valkyrie study, included very old patients with increased Agatston score (where arteries are in an advanced calcification non-reversable state) (De Vriese et al., 2020). Moreover, dose-finding studies have indicated that even doses as high as 460 μg/day of MK-7 are insufficient to fully correct vitamin K deficiency in hemodialysis patients, suggesting that higher doses (>500 μg/day) are necessary (Caluwé et al., 2014; Westenfeld et al., 2012).

To address these limitations, ongoing trials such as the UCASAL-VITK study (NCT04539418) and the VIKIPEDIA study (NCT04900610) are investigating the effects of higher MK-7 doses in hemodialysis (2,000 μg iv thrice weekly) and peritoneal dialysis (1,000 μg/day) patients, respectively (Roumeliotis et al., 2022b). The results of these trials are expected to provide crucial insights into the safety, tolerability, and efficacy of optimal MK-7 doses in uremic populations.

## Conclusion

Vascular calcification and arterial stiffness may represent pathophysiological mechanisms underlying the onset and progression of cognitive decline. Vitamin K deficiency is a key determinant of arterial health and, by extension, may influence cognitive function in the elderly. To elucidate the potential therapeutic benefits of MK-7 supplementation on cognitive function, future RCTs are needed. These trials should focus on using optimal dosages (>500 μg/day), ensuring long follow-up periods, and utilizing the most bioactive form of vitamin K (MK-7). Such studies are essential to establish whether vitamin K2 supplementation can play a role in preventing or mitigating cognitive decline in aging populations.
